# Clinical Experience of Craniospinal Irradiation Using Volumetric Modulated Arc Therapy on the Siddharth-II Linear Accelerator: A Dosimetric Analysis

**DOI:** 10.7759/cureus.115195

**Published:** 2026-08-25

**Authors:** Debnarayan Dutta, Dhiman Das, Paramita Sil, Avinash Kuppa Rao, Tamilarasan Mani, Dhivya S

**Affiliations:** 1 Radiation Oncology, 3D Medicare Private Limited, Bardhaman, IND; 2 Research and Development, Panacea Medical Technologies Pvt. Ltd., Bangalore, IND

**Keywords:** craniospinal irradiation (csi), dosimetric evaluation, o-ring type linear accelerator, quality assurance (qa), vmat csi

## Abstract

Aim: Craniospinal irradiation (CSI) is a technically challenging radiotherapy procedure due to the extended target volume, multiple isocenters, and the need to maintain dose uniformity across junction regions. This study evaluated the feasibility, workflow efficiency, and dosimetric performance of volumetric modulated arc therapy (VMAT)-based CSI delivered using the Siddharth-II O-ring gantry linear accelerator.

Materials and methods: A retrospective feasibility study was conducted in two patients with cerebral germinoma, treated with a multi-isocenter VMAT-based CSI technique on the Siddharth-II linear accelerator (Bangalore, India: Panacea Medical Technologies Pvt. Ltd.). Treatment planning was carried out in RayStation 12A (Stockholm, Sweden: RaySearch Laboratories) using 6 MV photon beams with three isocenters covering cranial, upper spinal, and lower spinal regions. Junction optimization was achieved using longitudinal overlap regions. Dosimetric evaluation included target coverage parameters (D98, D50, D2), conformity index (CI), homogeneity index (HI), and organ-at-risk (OAR) doses. Treatment delivery efficiency was assessed using monitor units and beam-on time. Patient-specific quality assurance was performed using a 2D detector array with 3%/3 mm gamma analysis. Cone-beam CT-based image guidance and six-degrees-of-freedom couch corrections were used for setup verification.

Results: Both patients achieved satisfactory target coverage with D98 values of 22.56 Gy and 24.29 Gy and D2 values of 24.19 Gy and 25.77 Gy, respectively. CI and HI values were 1.15 and 1.04, demonstrating favorable conformity and homogeneity. Junction regions showed smooth dose transitions without clinically significant hot or cold spots. Mean lung doses remained below 5 Gy, kidney doses below 6 Gy, and lens doses below 10 Gy. Gamma passing rates exceeded 98%, while treatment delivery times were approximately 8-9 min with total monitor units below 940.

Conclusion: VMAT-based CSI using the Siddharth-II O-ring gantry linear accelerator is feasible and demonstrates favorable dosimetric performance, effective junction management, reliable quality assurance, and efficient treatment delivery. These findings support the clinical applicability of this platform for complex craniospinal irradiation techniques.

## Introduction

Craniospinal irradiation (CSI) remains a key component in the treatment for central nervous system malignancies such as medulloblastoma and other embryonal tumors [1,2]. Owing to the extensive longitudinal target volume, achieving homogeneous dose delivery while minimizing exposure to surrounding organs-at-risk (OARs) remains challenging. Conventional CSI techniques based on matched radiation fields are associated with junction uncertainties and dose inhomogeneity that may result in clinically relevant hot and cold spots [3]. Earlier three-dimensional conformal radiotherapy (3D-CRT) approaches have also shown limitations in normal-tissue sparing and potential for increased treatment-related toxicity [4]. Advanced radiotherapy techniques, including intensity-modulated radiotherapy (IMRT) and volumetric modulated arc therapy (VMAT), have been developed to address these limitations, improving conformity, homogeneity, and OAR sparing [5,6]. VMAT enables continuous beam modulation during gantry rotation, facilitating highly conformal dose delivery with improved treatment efficiency [7]. Several studies have demonstrated dosimetric advantages of VMAT-based CSI, including improved planning target volume (PTV) coverage and reduced dose to critical structures such as the lungs, heart, and kidneys [8-10]. VMAT also facilitates multi-isocenter approaches necessary for coverage of the entire craniospinal axis [11]. An important consideration in VMAT-based CSI is optimization of junction regions, where overlap strategies and dose feathering have been shown to reduce dose gradients and improve robustness against setup uncertainties [12]. Despite these advances, most clinical reports have focused on widely established treatment platforms. The Siddharth-II (Bangalore, India: Panacea Medical Technologies Pvt. Ltd.) linear accelerator, an indigenously developed O-ring gantry-based system, has been designed to support advanced radiotherapy delivery with broader clinical accessibility and workflow efficiency. While volumetric modulated arc therapy (VMAT) is now widely implemented across modern linear accelerators, the successful execution of technically demanding procedures such as CSI requires not only VMAT capability but also precise mechanical stability, reproducible image guidance, accurate multi-isocenter coordination, and reliable junction management. The Siddharth-II combines VMAT delivery with a rigid wide-bore O-ring gantry architecture that provides enhanced mechanical stability and reduced gantry sag, thereby supporting accurate isocentric performance during long-field treatments. These characteristics are particularly important in CSI, where treatment delivery involves extended target volumes, multiple isocenters, and precise overlap of junction regions. The system further integrates image-guided radiotherapy (IGRT) capabilities, including dual-orthogonal kilovoltage imaging and cone-beam CT (CBCT)-based verification, together with robotic six-degrees-of-freedom (6-DOF) couch corrections, enabling reproducible patient setup and alignment throughout the craniospinal axis. In addition to routine VMAT treatments, these integrated capabilities facilitate the implementation of complex techniques such as multi-isocenter VMAT-based CSI with effective junction optimization and conformal target coverage. The compact system design and integrated beam stopper further contribute to efficient clinical deployment and reduced infrastructure requirements, making the platform suitable for advanced radiotherapy applications in routine clinical practice. In this context, the present study reports our initial institutional clinical experience in two patients, demonstrating the feasibility of VMAT-based craniospinal irradiation on Siddharth-II using a reproducible multi-isocenter approach with effective junction management. To our knowledge, this represents one of the initial reports describing VMAT-based CSI implemented on the Siddharth-II platform.

## Materials and methods

Patient selection and simulation

This retrospective feasibility study included two patients diagnosed with cerebral germinoma who underwent CSI using a VMAT technique on the Siddharth-II Iconic Plus linear accelerator (Bangalore, India: Panacea Medical Technologies Pvt. Ltd.), an O-ring gantry-based system. Patient prescriptions were as follows: patient 1 - 23.4 Gy in 13 fractions + 10.8 Gy boost in seven fractions; patient 2 - 25.2 Gy in 14 fractions + 9 Gy boost in five fractions.

Patient and treatment characteristics are summarized in Table 1. Patient immobilization was individualized according to clinical requirements. Patient 1 was immobilized using a brain thermoplastic mask in combination with a Vac-Lok cushion extending from the neck to the lower body. Patient 2 was immobilized with a head-and-neck thermoplastic mask and a pelvic thermoplastic mask [13]. CT simulation was performed from the vertex to the sacrum using 2.5 mm slice thickness. Images were transferred to RayStation 12A (Stockholm, Sweden: RaySearch Laboratories) for contouring and planning.

**Table 1:** Patient and treatment characteristics. CSI: craniospinal irradiation

Patient (age), years/sex	Diagnosis	CSI prescription	Boost dose	CSI isocenters
13/F	Cerebral germinoma	23.4 Gy/13 fx	10.8 Gy/7 fx	3
22/M	Cerebral germinoma	25.2 Gy/14 fx	9 Gy/5 fx	3

Target delineation and organs-at-risk

The clinical target volume included the whole brain, meninges, and spinal canal. Planning target volume (PTV) margins of 3 mm and 5 mm were applied for patients 1 and 2, respectively. Organs-at-risk (OARs) contoured for optimization and evaluation included the brainstem, optic apparatus, eyes, lenses, cochlea, pituitary, thyroid, lungs, heart, liver, kidneys, and other relevant normal tissues. Contouring was performed in accordance with standard recommendations and institutional guidelines [14,15].

Treatment planning technique

**Figure 1:**
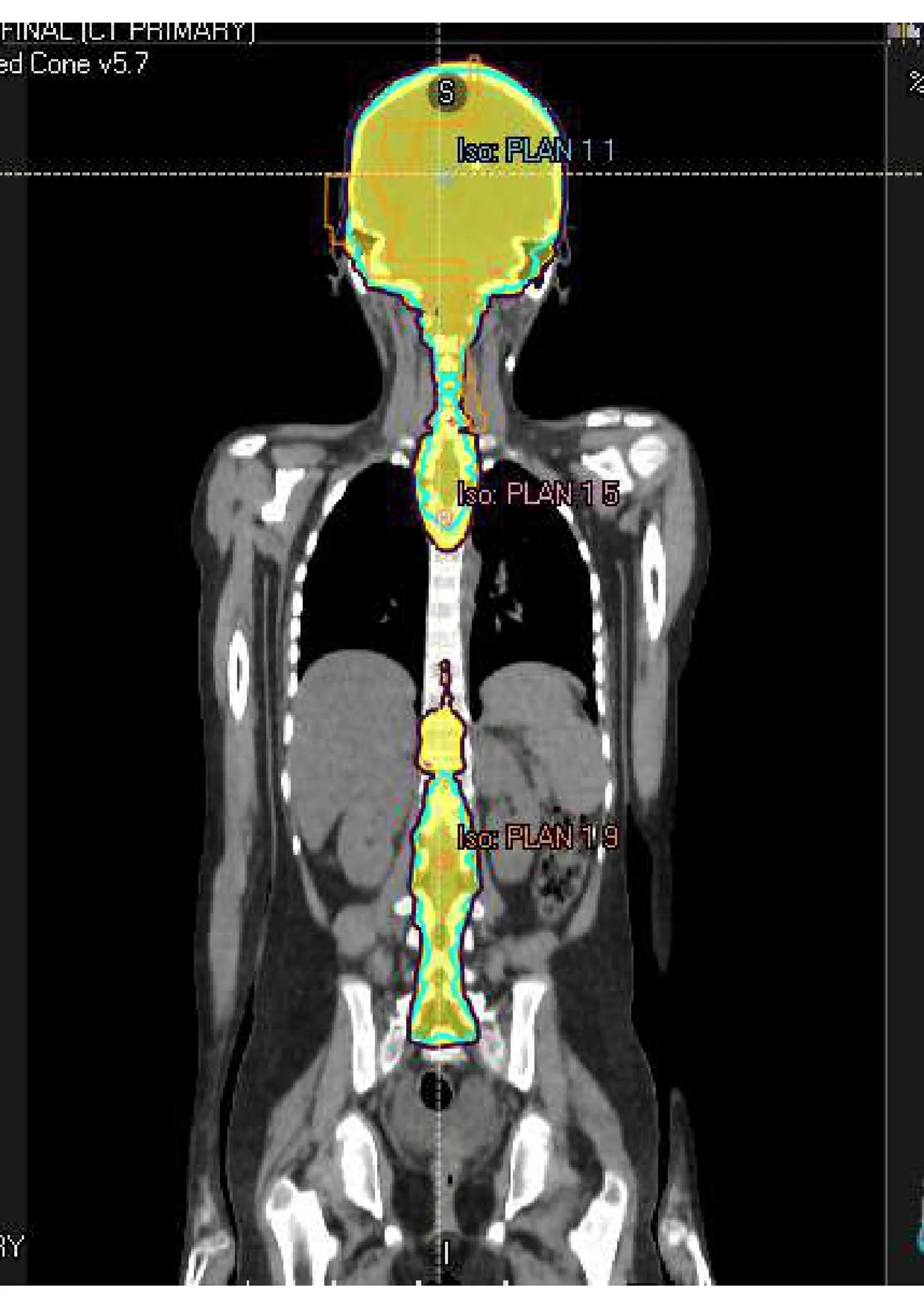
Multi-isocenter VMAT arrangement demonstrating cranial and spinal isocenter placement for patient 1. VMAT: volumetric modulated arc therapy

**Figure 2:**
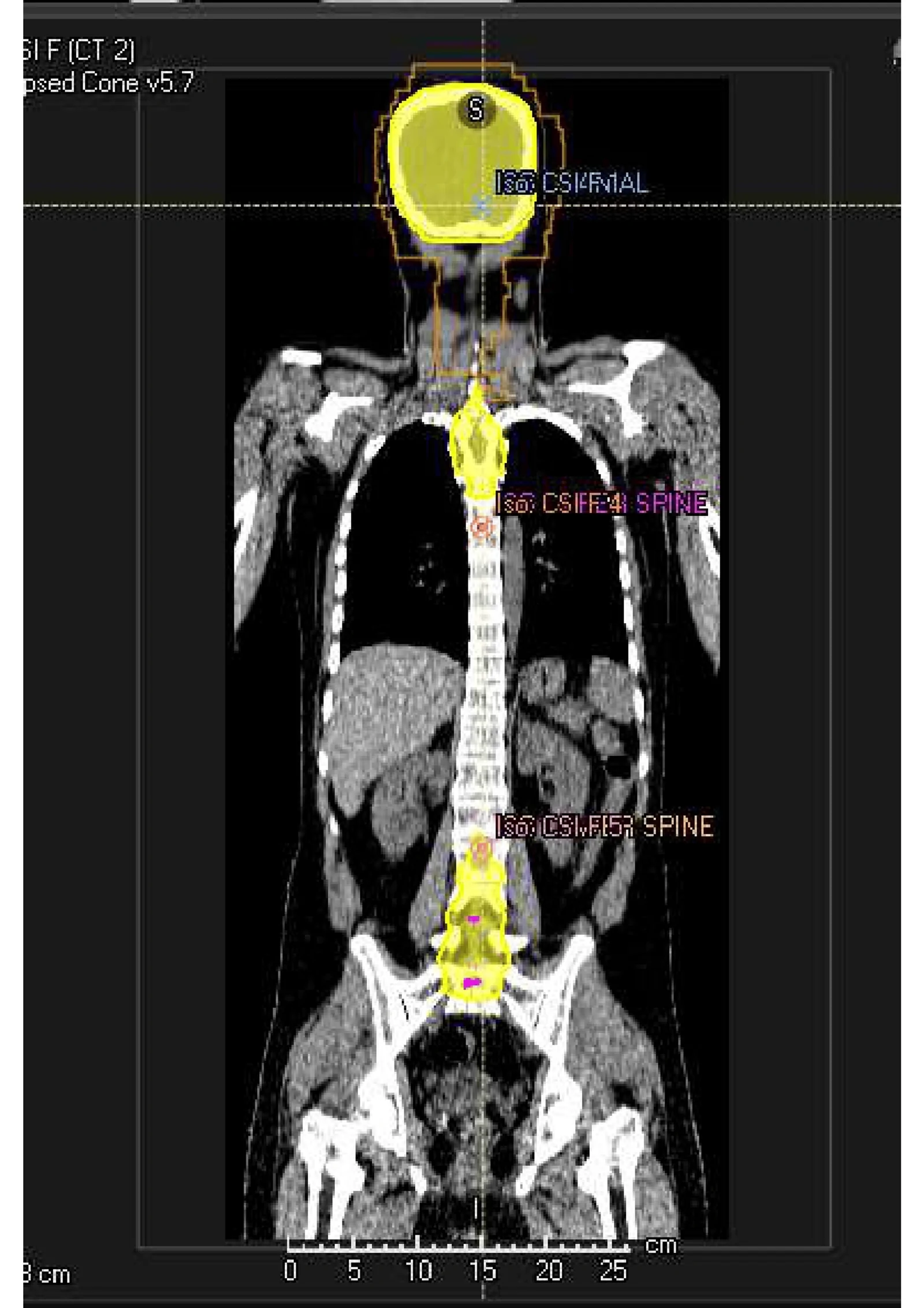
Multi-isocenter VMAT arrangement demonstrating cranial and spinal isocenter placement for patient 2. VMAT: volumetric modulated arc therapy; CSI: craniospinal irradiation

Treatment planning was performed using RayStation 12A (Stockholm, Sweden: RaySearch Laboratories), with dose calculations carried out using the collapsed cone algorithm. All treatment plans were delivered on the Siddharth-II Iconic Plus linear accelerator (Bangalore, India: Panacea Medical Technologies Pvt. Ltd.) using 6 MV photon beams. This machine is equipped with a 30-leaf-pair multi-leaf collimator with a leaf width of 10 mm at isocenter and a maximum field size of 30×30 cm². VMAT planning was performed using a three-isocenter arrangement comprising cranial, upper spinal, and lower spinal isocenters. The multi-isocenter VMAT arrangement for both patients is illustrated in Figures 1, 2.

The cranial isocenter was planned using two coplanar full arcs (360° each) to ensure adequate modulation of the cranial target volume. Each spinal isocenter was planned with four partial arcs. For the upper spinal isocenter, the arcs spanned 181°-261° and 71°-179°, each delivered in both clockwise (CW) and counterclockwise (CCW) directions. Similarly, the lower spinal isocenter was treated with four partial arcs spanning 181°-271° and 71°-179°, each delivered in both CW and CCW directions. A skip sector from 272° to 70° was applied for both spinal isocenters. This design was intentionally selected to favor posterior beam entry, thereby minimizing low-dose exposure to anterior organs-at-risk such as the heart, lungs, and bowel, while still providing adequate modulation to ensure complete target coverage. Longitudinal overlap regions of 9.51 cm for patient 1 and 4.94 cm for patient 2 were incorporated to maintain smooth dose transitions across junctions.

Planning objectives included achieving at least 95% PTV coverage, limiting the maximum dose to 115% of the prescribed dose, controlling hotspot formation, and minimizing dose to organs-at-risk. Plan evaluation was based on dose-volume parameters including D98, D50, D2, as well as conformity and homogeneity indices [5,16,17]. The total number of monitor units was 939 and 928 for patients 1 and 2, respectively, with treatment delivery times of 516 s and 500 s.

Dosimetric evaluation

Conformity index (CI) was calculated using Paddick’s formulation [16]. The homogeneity index (HI) was calculated as the ratio of D5% to D95% [17]. Dose-volume histograms were used for target and organ-at-risk evaluation.

Quality assurance and treatment verification

Patient-specific quality assurance was performed for all treatment plans prior to delivery using the Raydose 2DMap detector array (Guangzhou, China: Guangzhou Raydose Medical Technology Co., Ltd.), a planar detector array designed for patient-specific VMAT verification. Agreement between measured and calculated dose distributions was evaluated using 3%/3 mm gamma analysis [18]. Image guidance using cone-beam CT (CBCT) was performed before each treatment fraction to verify the isocenter. Online image registration was carried out, followed by couch corrections using the six-degrees-of-freedom (6-DOF) couch to ensure accurate and reproducible patient positioning throughout the treatment course [19,20].

## Results

Target coverage and dose distribution

Adequate target coverage was achieved in both cases, with D98 values of 22.56 Gy and 24.29 Gy and D2 values of 24.19 Gy and 25.77 Gy, respectively. The dose received by 95% of the PTV (D95) was 97.73% and 99.90% of the prescribed dose for patients 1 and 2, respectively. Median target dose (D50) values were 23.44 Gy and 25.33 Gy. Volumetric dose coverage analysis demonstrated V95% values of 98.0% and 99.15%, respectively.

**Figure 3:**
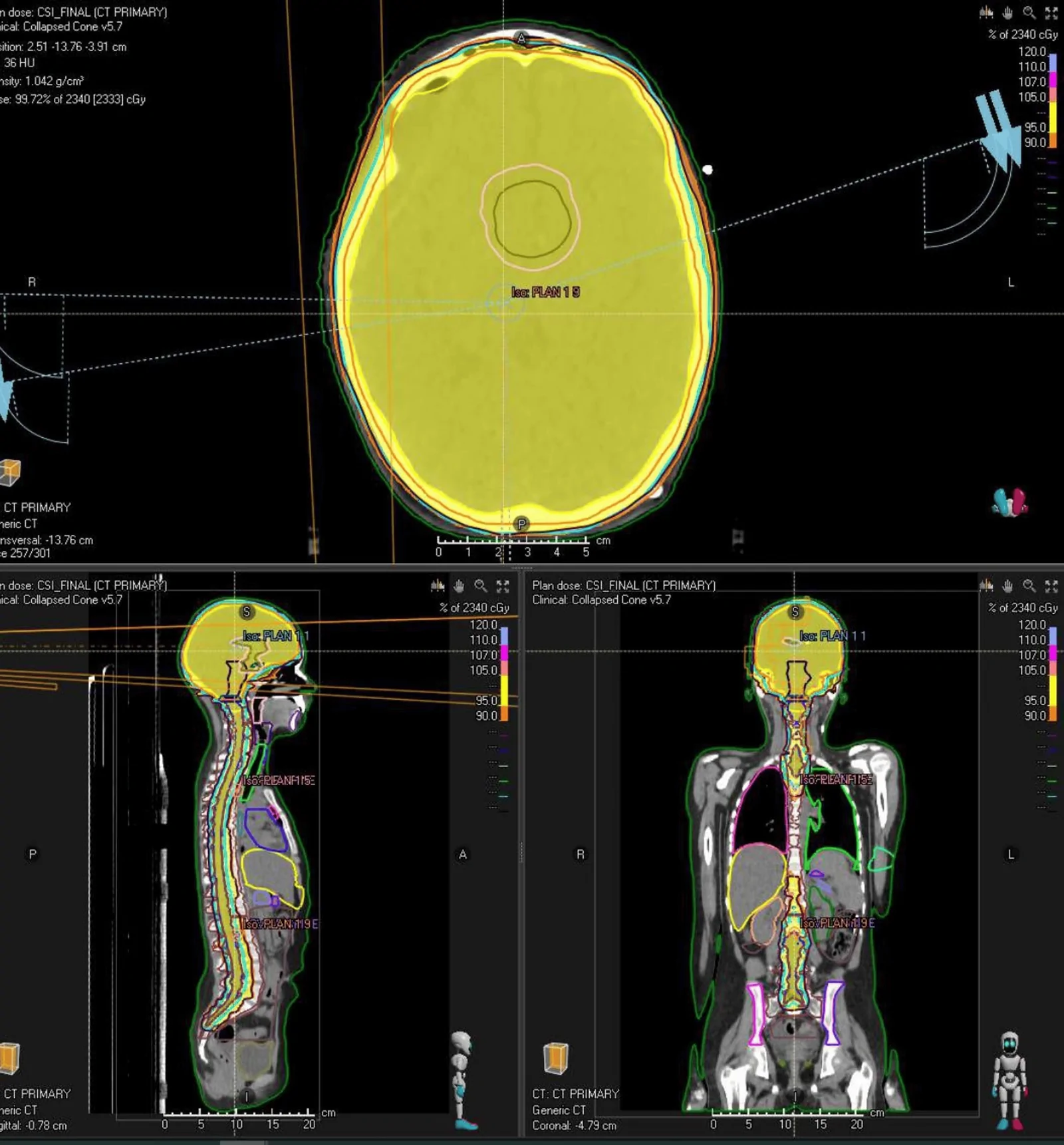
Representative axial, sagittal, and coronal dose distributions for VMAT-based craniospinal irradiation in patient 1. VMAT: volumetric modulated arc therapy; CSI: craniospinal irradiation

**Figure 4:**
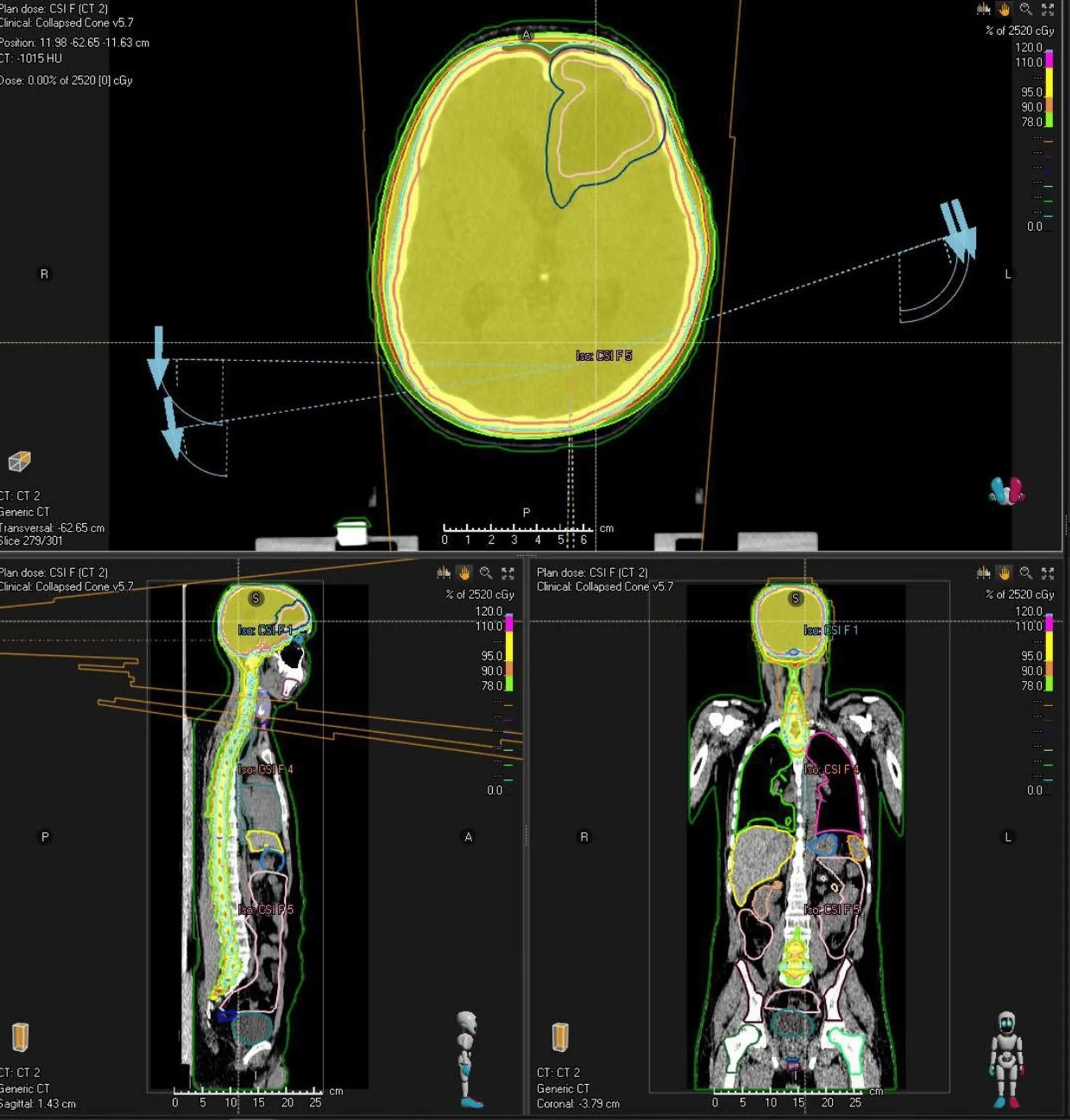
Representative axial, sagittal, and coronal dose distributions for VMAT-based craniospinal irradiation in patient 2. VMAT: volumetric modulated arc therapy; CSI: craniospinal irradiation

**Figure 5:**
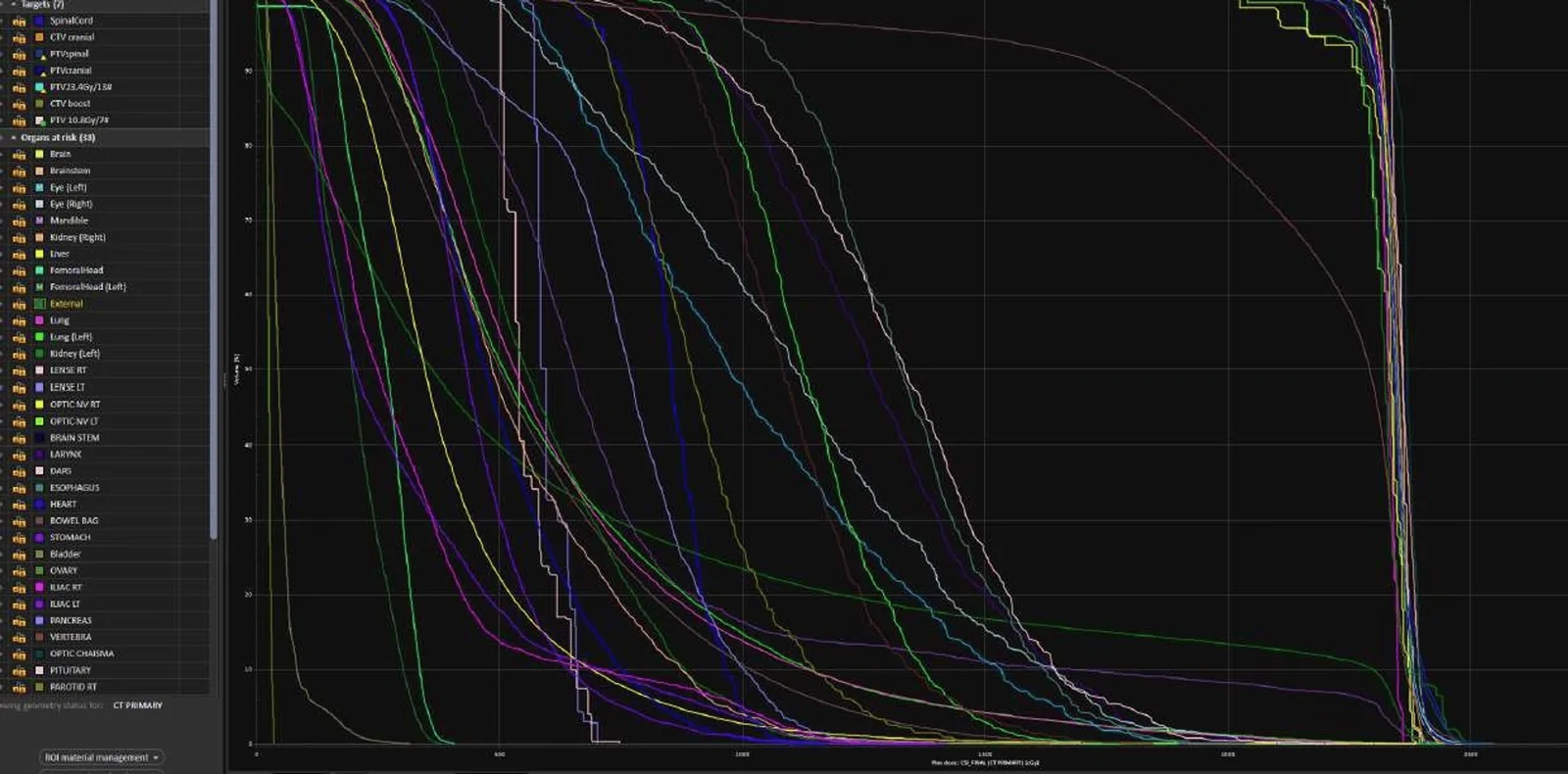
Dose-volume histograms (DVHs) for target volumes and major organs-at-risk for patient 1.

**Figure 6:**
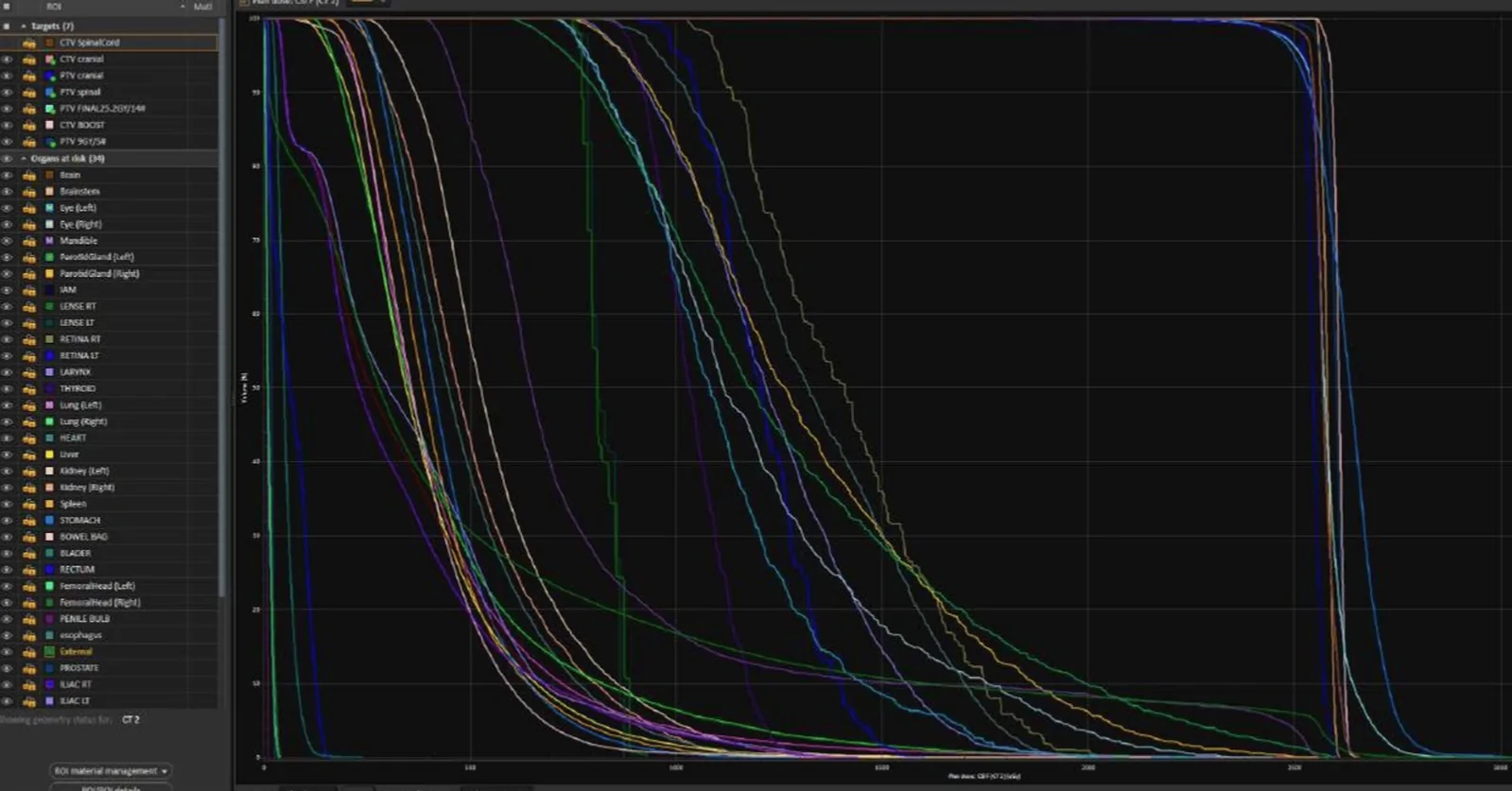
Dose-volume histograms (DVHs) for target volumes and major organs-at-risk for patient 2.

High-dose regions were limited, with V107% values of 0.03% and 3.07%, while V110% remained negligible at 0% and 0.65%, indicating acceptable hotspot control. Representative dose distributions are shown in Figures 3, 4, and corresponding dose-volume histograms are presented in Figures 5, 6.

Dosimetric indices

The conformity index (CI) was 1.15 for both patients, while the homogeneity index (HI) was 1.04 for both plans. Target dosimetric parameters are summarized in Table 2.

**Table 2:** Target dosimetric parameters (CSI phase only, excluding boost). CSI: craniospinal irradiation; CI: conformity index; HI: homogeneity index

Parameters	Patient 1	Patient 2
D98 (Gy)	22.56	24.29
D50 (Gy)	23.44	25.33
D2 (Gy)	24.19	25.77
D95 (% of prescribed dose)	97.73	99.90
CI	1.15	1.15
HI	1.04	1.04

Junction management

Junction management remains a critical component of CSI planning because dose gradients at field overlaps can lead to clinically significant over- or underdosage. In this study, overlap regions of 9.51 cm and 4.94 cm between adjacent isocenters produced smooth dose transitions without significant hotspots or cold spots. Junction quality was evaluated by reviewing the axial, sagittal, and coronal dose distributions together with dose-volume histograms, with particular emphasis on the overlap regions to assess dose uniformity and identify hotspot and cold spot formation. These observations align with prior reports showing that overlap-based junction strategies improve robustness against setup uncertainties and reduce sensitivity to longitudinal shifts [12]. The favorable junction performance observed supports the effectiveness of the adopted multi-isocenter overlap approach and addresses one of the traditional limitations of craniospinal irradiation.

Organs-at-risk sparing

Organ-at-risk dosimetric parameters are summarized in Table 3. Mean lung doses were 4.34 Gy and 4.45 Gy, respectively. Mean kidney doses remained below 6 Gy in both patients, while lens doses were maintained below 10 Gy. Doses to the optic apparatus, thyroid, heart, liver, and other critical structures remained within clinically acceptable limits.

**Table 3:** Dosimetric parameters for organs-at-risk for CSI and intracranial boost. CSI: craniospinal irradiation, L: left; R: right

Organ-at-risk	Metric	Patient 1 (Gy)	Patient 2 (Gy)
Brainstem	D_max _	34.20	34.40
Optic nerve (L)	D_max _	30.65	29.50
Optic nerve (R)	D_max _	29.90	29.80
Optic chiasm	D_max _	34.80	34.20
Eye (L)	D_max _	22.90	28.30
Eye (R)	D_max _	23.60	19.90
Lens (L)	D_max _	9.33	8.80
Lens (R)	D_max _	8.79	8.90
Cochlea (L)	D_max _	26.30	25.90
Cochlea (R)	D_max _	26.70	26.80
Pituitary	D_max _	23.85	24.81
Thyroid	D_mean_	8.99	10.42
Total lung	D_mean_	4.34	4.45
Heart	D_mean_	4.47	4.94
Liver	D_mean_	4.06	3.95
Left kidney	D_mean_	5.40	5.98
Right kidney	D_mean_	5.90	5.15
Larynx	D_mean_	12.80	12.36
Bladder	D_mean_	5.50	5.30
Femoral head (L)	D_mean_	2.56	12.0
Femoral head (R)	D_mean_	2.0	13.0

Clinical feasibility and implementation

**Figure 7:**
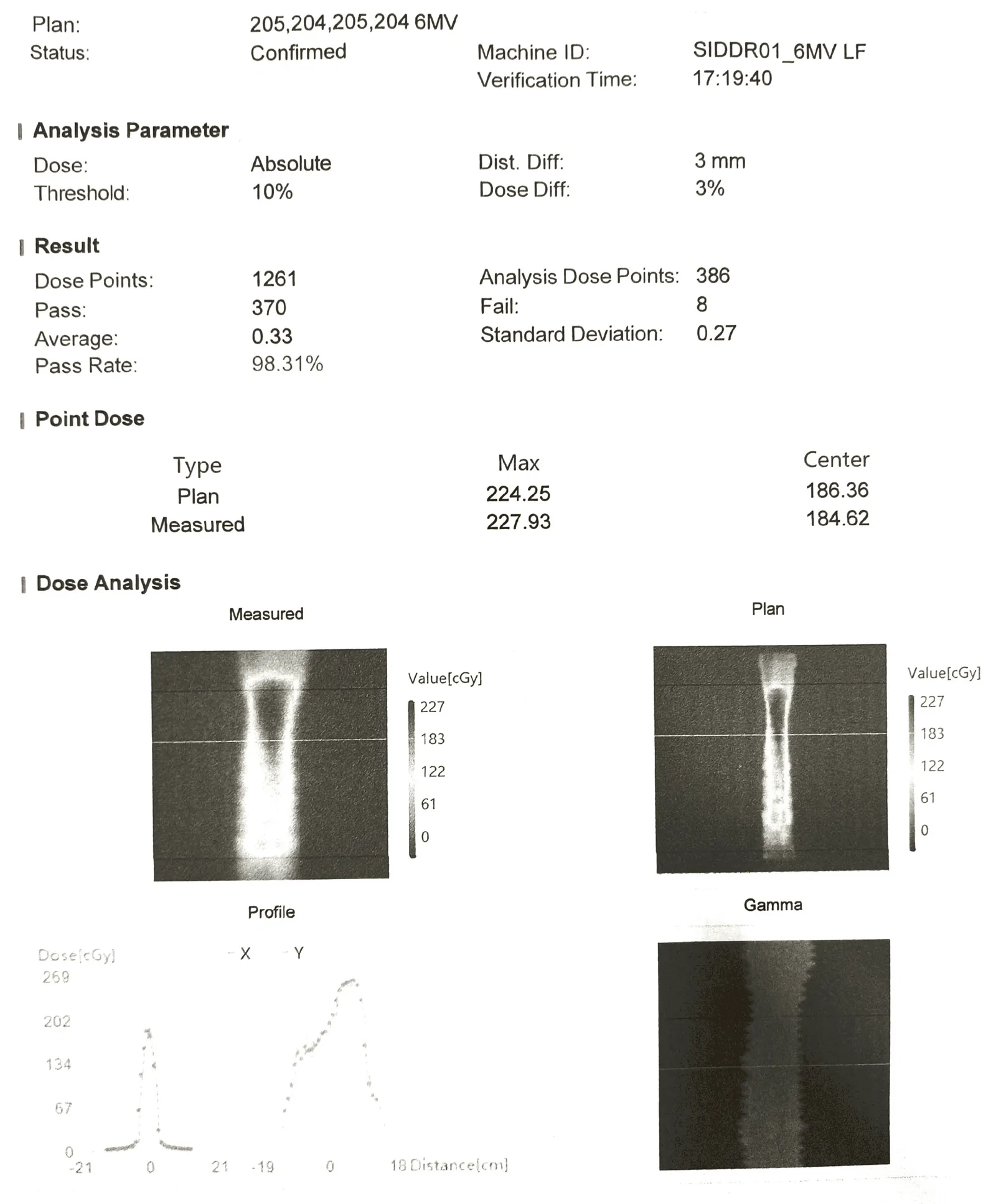
Gamma analysis for patient 1.

**Figure 8:**
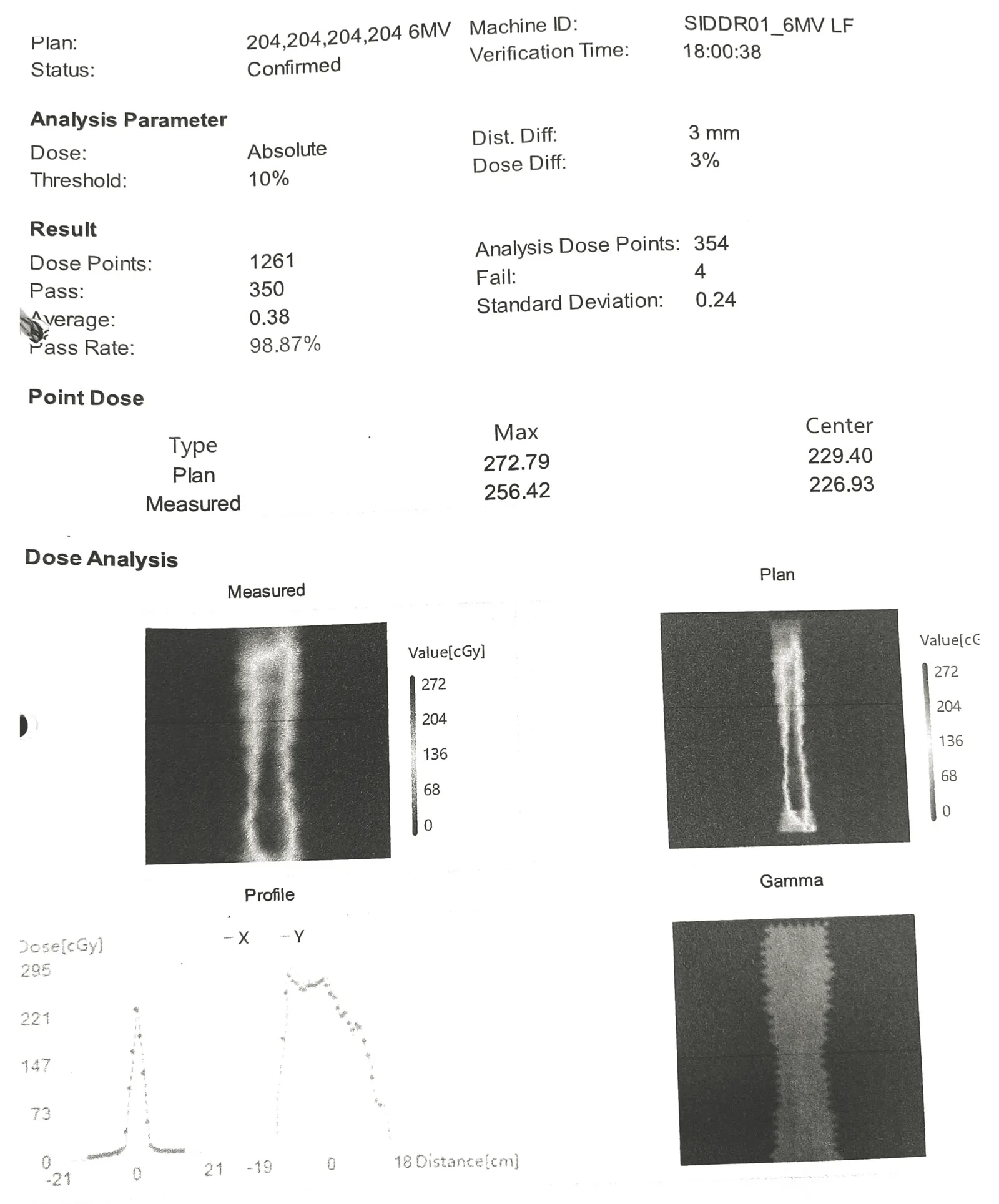
Gamma analysis for patient 2.

Patient-specific pretreatment dose verification was performed by comparing the calculated treatment plans with the corresponding measured dose distributions. The analysis used absolute dose comparison with a 3% dose-difference and 3 mm distance-to-agreement criterion and a 10% dose threshold. The measured and planned dose distributions, profiles, and gamma maps demonstrated good spatial and dosimetric agreement for both treatment plans, with gamma passing rates of 98.31% and 98.87% for patients 1 and 2, respectively. Representative gamma analysis results for both patients are shown in Figures 7, 8. These findings, together with the favorable dosimetric performance, support the practical feasibility of implementing VMAT-based CSI on the Siddharth-II platform for complex multi-isocenter treatments. Comparable studies have similarly emphasized the importance of image guidance and delivery verification for reliable CSI implementation [18-20].

Study significance and limitations

The present study is limited by the small sample size and the absence of long-term clinical outcome evaluation. The findings therefore primarily reflect the technical feasibility and dosimetric performance of VMAT-based craniospinal irradiation on the Siddharth-II platform. In addition, patient-specific dosimetric variations were observed, including differences in hotspot volume within the junction regions and femoral head dose. Although CI and HI were identical for the two independently optimized treatment plans, these values were verified and confirmed to be correct. The limited sample size does not permit further interpretation of whether this reflects the planning methodology, comparable target characteristics, or a coincidental finding. Similarly, while patient 2 demonstrated a larger hotspot volume despite a shorter junction overlap, the present feasibility study does not permit a definitive assessment of the relationship between junction overlap and hotspot volume.

## Discussion

Target coverage and plan quality

The achieved target coverage and dose homogeneity in the present study were comparable to previously reported VMAT-based CSI series. Fogliata et al. demonstrated improved conformity and a more homogeneous dose distribution with VMAT for CSI compared with conventional approaches [5]. Similar findings were reported by Lee et al. and Sarkar et al., who observed favorable target coverage and reduced dose to normal tissues with VMAT-based techniques [8,9].

Junction management

Junction management remains one of the major challenges in CSI because small setup uncertainties can produce significant dose variations at field overlaps. Previous investigations have shown that overlap-based junction strategies improve robustness and reduce steep dose gradients across adjacent treatment regions [12,13]. The smooth junction dose transitions observed in the present study are consistent with these reports.

Organ-at-risk sparing

VMAT-based CSI has been associated with improved organ-at-risk sparing compared with conventional CSI and fixed-field intensity-modulated radiotherapy (IMRT) techniques [5,8-10]. In the present study, mean lung and kidney doses remained low, supporting the ability of VMAT optimization to limit unnecessary irradiation of normal tissues.

Clinical feasibility of Siddharth-II

Successful CSI delivery requires accurate image guidance, reproducible patient positioning, and reliable multi-isocenter coordination. Previous reports have emphasized the importance of image-guided verification and the accuracy of treatment delivery in CSI workflows [18-20]. The favorable QA results and reproducible treatment setup observed in the present study further support the feasibility of implementing VMAT-based CSI on the Siddharth-II platform.

Limitations

The present study is limited by the small sample size, absence of long-term clinical outcome evaluation, and short follow-up duration. The findings therefore primarily reflect technical feasibility and dosimetric performance. Nevertheless, the consistency of target coverage, organ-at-risk sparing, and quality assurance results supports the feasibility of VMAT-based CSI delivery on Siddharth-II. Larger prospective studies with clinical outcome analysis are warranted.

## Conclusions

VMAT-based craniospinal irradiation using the Siddharth-II linear accelerator demonstrated favorable dosimetric performance, with satisfactory target coverage, good dose homogeneity, and effective sparing of organs-at-risk. The multi-isocenter approach with optimized junction management enabled smooth dose transitions across the craniospinal axis, supporting robust and reliable treatment delivery. This initial institutional clinical experience highlights the potential of Siddharth-II to deliver advanced radiotherapy techniques such as VMAT-based CSI. Further studies with larger patient cohorts are warranted to validate these findings.
